# Functional Characteristics of the Naked Mole Rat μ-Opioid Receptor

**DOI:** 10.1371/journal.pone.0079121

**Published:** 2013-11-27

**Authors:** Melanie Busch-Dienstfertig, Clarisse A. Roth, Christoph Stein

**Affiliations:** Department of Anesthesiology and Intensive Care Medicine, Charité Campus Benjamin Franklin, Freie Universität Berlin, Berlin, Germany; BioScience Project, United States of America

## Abstract

While humans and most animals respond to µ-opioid receptor (MOR) agonists with analgesia and decreased aggression, in the naked mole rat (NMR) opioids induce hyperalgesia and severe aggression. Single nucleotide polymorphisms in the human mu-opioid receptor gene (*OPRM1*) can underlie altered behavioral responses to opioids. Therefore, we hypothesized that the primary structure of the NMR MOR may differ from other species. Sequencing of the NMR *oprm1* revealed strong homology to other mammals, but exposed three unique amino acids that might affect receptor-ligand interactions. The NMR and rat *oprm1* sequences were cloned into mammalian expression vectors and transfected into HEK293 cells. Radioligand binding and 3'-5'-cyclic adenosine monophosphate (cAMP) enzyme immunoassays were used to compare opioid binding and opioid-mediated cAMP inhibition. At normalized opioid receptor protein levels we detected significantly lower [3H]DAMGO binding to NMR compared to rat MOR, but no significant difference in DAMGO-induced cAMP inhibition. Strong DAMGO-induced MOR internalization was detectable using radioligand binding and confocal imaging in HEK293 cells expressing rat or NMR receptor, while morphine showed weak or no effects. In summary, we found minor functional differences between rat and NMR MOR suggesting that other differences e.g. in anatomical distribution of MOR underlie the NMR's extreme reaction to opioids.

## Introduction

The µ-opioid receptor (MOR) mediates the analgesic properties of MOR ligands (e.g. morphine), the oldest and most powerful analgesics [1]. *OPRM1*-like sequences have been present since the beginning of vertebrate evolution and remain highly homologous across a broad range of species [2]. Accordingly, the behavioral response to MOR agonists is also highly conserved and includes analgesia, sedation, and decreased aggression [3]. However, one exception is the naked-mole rat (NMR) which displays hyperactivity, motor dysfunction and, most notably, extreme aggression in response to MOR agonists [4,5]. These behaviors are reversible by naloxone (NLX), a non-selective opioid receptor antagonist, demonstrating that these effects are mediated by opioid receptor activation. Furthermore, morphine induced hyperalgesia in the hot-plate test (4) and much higher doses of opioids were required to produce analgesia in the formalin test compared to mice (5). The molecular basis for these unique reactions has not been examined so far.

 Studies have described dramatic effects of single nucleotide polymorphisms (SNPs) in the 5’-end of *OPRM1*. The most prominent is the N40D mutation (A118G), which is naturally occurring in up to 50% of humans and is associated with increased consumption of analgesics [6–9]. Multiple studies have described a similar SNP in rhesus macaque and in a mouse model of the human SNP, both of which mimic the behavioral alterations to opioids occurring in humans carrying this SNP [10,11]. Interestingly, these SNPs are also associated with heightened aggression, stronger maternal bonding, and increased social attachment [12–15], and several *in-vivo* [16,17] and *in-vitro* [18–20] studies examining the effects of *OPRM1* SNPs show significant changes in ligand binding and receptor activation

 Because of the behavioral alterations associated with SNPs in the MOR gene, we hypothesized that the reactions to opioids observed in the NMR might be associated with amino acid (aa) alterations in MOR. Therefore, we sequenced the *oprm1* of the NMR in order to compare its primary structure to those of other species. To test the hypothesis that a unique aa alteration in the NMR MOR alters its function, we cloned the receptor into a bicistronic mammalian expression vector for analysis of radioligand binding, receptor endocytosis and MOR-mediated 3’-5’-cyclic adenosine monophosphate (cAMP) repression. 

## Materials and Methods

### Agreed ethics statement

Tissue used to isolate RNA or DNA was obtained from animals that died of natural causes. All tissue used in the study was obtained from animals housed in two breeding colonies at the Leibniz Institute for Zoo and Wildlife Research, Berlin, Germany and was donated by Dr. Thomas Hildebrandt.

### Primer synthesis

Primers were designed based on rat, mouse and guinea pig *oprm1* sequences published on PubMed nucleotide (Accession numbers NM_O13071, U26915, and NM_001172738, respectively). A homology map of these *oprm1* sequences using the program PRALINE (Amsterdam, the Netherlands) was created and areas with the most highly conserved regions were used to design primers. Since the NMR’s closest relative with a published *oprm1* sequence is the guinea pig, we based our final primers on the guinea pig *oprm1* sequence. Primers ranging from 12 to 26 base pairs were synthesized by TIB MOLBIOL Syntheselabor GmbH (Berlin, Germany). Over 30 different primers were tested and those with the closest match to the NMR sequence are listed in Table S1. 

### Polymerase chain reaction (PCR) and sequencing

PCR reactions were prepared with Thermo Scientific Phusion® DNA polymerase according to the manufacturer's instructions and at the following temperatures: After denaturation at 98°C for 30 s, 40 cycles with denaturation at 98°C for 10 s, primer annealing at 55 °C for 45 s, and elongation at 72°C for 45 s were performed. PCR products were run on a 1% agarose gel and single bands of interest were isolated using the Qiagen Extraction Kit. Purified products were sent for sequencing to AGOWA Genomics (Berlin, Germany). For sequence alignments and homology searches we utilized the www.ncbi.nlm.nih.gov database and A Plasmid Editor software. We published the entire coding sequence of the NMR *oprm1* online in the National Center for Biotechnology Information (Accession number JQ011280).

### 2D Protein prediction and sequence alignment

**Figure 1:**
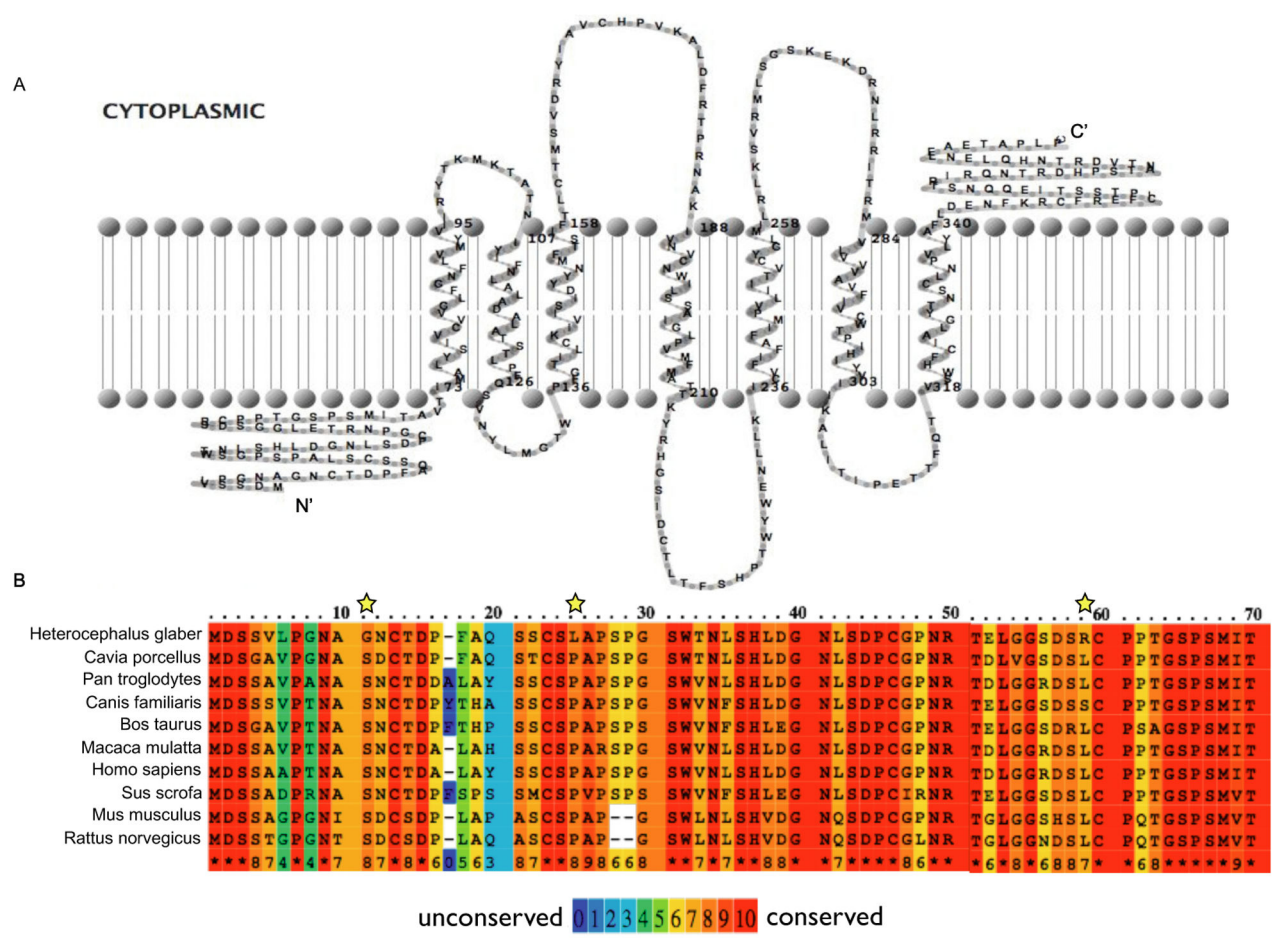
The NMR MOR N-terminus contains unique amino acids (aa) compared to other species. **A**) 2-D prediction of the NMR MOR secondary structure. **B**) The first 70 aa of the N-terminus of the NMR MOR are compared to 9 different mammalian species. Amino acid variations unique to the NMR compared to the other species are marked with asterisks.

The online program TMRPres2D [21] was used to predict the number of transmembrane domains in the NMR MOR and to create a 2D model (Figure 1A). The complete aa sequence of the NMR MOR was aligned with the MOR of 9 other species published on the NCBI website using the online tool PRALINE (http://www.ibi.vu.nl/programs/pralinewww, University of Amsterdam, The Netherlands, Figure 1 B). Accession numbers were: AEX59148 (NMR), NP_001166209 (Cavia porcellus), NP_001029087 (Pan troglodytes), XP_003432592 (Canis lupus familiaris), AAB49477 (Bos Taurus), AAF97249 (Macaca mulatta), AAH74927 (Homo sapiens), AAB53770 (Sus scrofa), AAI19546 (Mus musculus), NP_037203 (Rattus norvegicus)

### Cloning and mutagenesis

To create *oprm1*-containing vectors for transfection, we extended single restriction sites at the 5' and 3' ends of amplified *oprm1* and inserted the resulting amplicons into the pIRES2-eGFP (enhanced green fluorescent protein) vector directly downstream of the CMV-promoter. The transfection primers and restriction enzymes are listed in Table S2; vector maps for rat and NMR pCMV-*oprm1*-IRES-eGFP are provided in Text S1 and S2, respectively. For imaging of cellular localization, the IRES sequence separating *oprm1* from eGFP was removed using the QuickChange® site-directed mutagenesis kit (Agilent Technologies, California, USA) to create viable eGFP fusion proteins. Although the mutagenesis kit is mainly applied to replace one or few nucleotides, it can also be used to insert or excise larger sequences, as described by the manufacturer. Chimeric oligonucleotide primers were designed that were complementary to 4-5 codons of the *oprm1* 3’-end before the stop and of the eGFP 5’-end including the start site; primer sequences are given in Table S3. Extension of the oligonucleotide primers generated mutated plasmids without *oprm1* stop and IRES, but containing staggered nicks. Following amplification, the product was treated with Dpn I to remove the parental DNA template. The mutated vector DNA was then transfected into competent cells for nick repair. Colonies were tested for the absence of IRES using PCR, and NMR pCMV-*oprm1-*eGFP vector DNA (see Text S3 for vector map) was used for transfection and confocal imaging.

### Cell culture and transfection

HEK293 cells (DSMZ, Braunschweig, Germany) were cultured in Dulbeccos’s Modified Eagle’s Medium with 10% fetal bovine serum and 1% penicillin at 37°C with 5% CO_2_. One day prior to transfection roughly 2 x 10^6^ cells were seeded on 10 cm diameter (78.5 cm^2^) culture dishes. Cells were transiently transfected using FuGENE HD and EXTREME transfection reagent (Roche Applied Science) according to the manufacturer’s protocol, and at a ratio of 2:1 FuGENE to DNA. Cells were harvested for membrane preparations 24 h post transfection. Two dishes transfected with the same vector DNA were pooled for saturation binding experiments. For whole cell binding experiments approximately 0.6 x 10^6^ cells were grown and transfected in 25 cm^2^ cell culture bottles. Six bottles were transfected with the same transfection mixture in each experiment. For imaging, cells were grown on polylysine-coated glass in 6-well plates; nine wells were transfected with the same transfection mixture.

### Membrane preparations

Rat/NMR pCMV-*oprm1*-IRES-eGFP transfected HEK293 cells were washed twice with ice cold assay buffer (50 mM Tris-HCl, 1 mM EGTA, pH 7.4). Cells were then scraped from the culture dish in 10 ml ice cold assay buffer, homogenized with a Polytron homogenizer (Kinematica AG, Littau, Switzerland), and centrifuged at 42,000 g at 4°C for 20 min. Pellets were resuspended in assay buffer and homogenization was repeated twice. Subsequent to protein measurement the membranes were aliquoted and stored at -80°C. 

Cell lysates were processed using a plasma membrane protein extraction kit (Abcam) following the manufacturers' instructions. Cells were lysed by repeated freezing (liquid nitrogen) and thawing (37°C water bath) in homogenization buffer supplemented with protease inhibitors. Debris was removed by centrifugation (700 g for 10 min at 4°C). Total membrane protein was isolated from the cytosol fraction by high speed centrifugation of the supernatants (10,000 g for 30 min at 4°C). Pellets containing proteins from both plasma membrane and cellular organelle membranes were then resuspended in upper phase solution, mixed with lower phase solution, and centrifuged at 1,000 g for 5 min. The upper phase was collected and mixed for extraction with lower phase solution. After centrifugation (1,000 g for 5 min) the supernatant was harvested and diluted in water. Pellets obtained after a final centrifugation at top speed in a microcentrifuge (10 min, 4°C) were used for radioligand binding.

### Radioligand saturation binding assay

Tritium-tagged DAMGO ([D-Ala2, N-MePhe4, Gly-ol]-enkephalin, Perkin Elmer) was used as previously described [22]. Total binding was determined using approximately 100 µg of membrane protein for each concentration of [3H]DAMGO (1, 2, 4, 8, 16 and 32 nM). Non-specific binding was determined at each concentration of [3H]DAMGO by using 10 µM NLX. All measurements were performed in duplicate. Specific binding was calculated as the difference between the counts per minute (CPM) of total and non-specific binding. In addition, binding to non-transfected HEK293 cells was determined. This showed no differences between total and non-specific binding. The specific CPM were divided by the specific activity of [3H]DAMGO (81.7 CPM/fmol) to calculate the amount of bound ligand at each concentration in fmol/mg of total protein. A non-linear regression one-site binding model provided by GraphPad Prism was fit to each construct measured in each experiment in order to calculate the asymptote (Bmax) or maximum amount of bound ligand in fmol. The amount of MOR in each transfection was then calculated from the Bmax. Assuming that one molecule of ligand binds to one molecule of MOR, and a Hill slope of 1, the total amount of receptor per sample was calculated in μg. The molecular mass of each receptor was predicted based on its aa sequence (NIH Acession numbers NP_037203 for the rat MOR and AEX59148 for the NMR MOR) using the online Protein Mass Calculator (University of Leeds, United Kingdom). The molecular mass was 44.5 kDa for the rat and 44.8 kDa for the NMR MOR. Binding data were normalized by dividing the specific CPM values at each ligand concentration by the amount of MOR (in μg) in that sample. Using GraphPad Prism 4.0c, these normalized values were plotted and fit with a non-linear regression one-site binding model to determine Kd values. The mean area under the curve (AUC) for each construct was calculated.

### Radioligand binding to plasma membrane

To determine if the MORs expressed in transfected HEK293 cells were inserted into the cell membrane, plasma membrane extracts were subjected to [3H]DAMGO binding. Only one concentration (16 nM) of radioligand was tested. Non-specific binding was determined by the addition of 10 µM NLX. 

### Whole cell radioligand binding

Whole cell [3H]DAMGO binding was performed to determine receptor internalization according to Evans’ protocol [23]. Rat/NMR pCMV-*oprm1*-IRES-eGFP transfected HEK293 cells were exposed for 30 min at 37°C to 10 µM untagged DAMGO (Sigma-Aldrich), 10 µM morphine-sulfate (Sigma-Aldrich) or to vehicle (HBSS buffer supplemented with NaHCO_3_). Another set of cells was pre-incubated with 0.4 M sucrose in HBSS buffer for 30 min at 37°C prior to agonist exposure to inhibit opioid receptor internalization as previously described [24]. Cells were washed twice with ice cold HBSS and incubated for 5 min at 4°C in low pH stripping buffer (0.2 M acetic acid, 0.5 M saline, pH 2.5) to remove surface-bound but not internalized ligands that could interfere with radioligand binding. Subsequent to two washing steps, the cells were harvested and resuspended in cold HBSS buffer (1.6 ml per cell culture bottle). 0.4 ml of the suspensions were exposed to 16 nM [3H]DAMGO (total binding) or 16 nM [3H]DAMGO plus 10 µM NLX (non-specific binding) in duplicates. This incubation was performed for 2 h at 4°C to promote binding of the radioligand to surface receptors and to prevent further receptor internalization. Cells were then transferred to 0.1% polyethyleneimine-presoaked GF/B filters (Whatman), unbound radioligand was removed by washing with 50 mM Tris buffer, and filters were incubated for 24 h in 3 ml scintillation liquid. Specific CPM were calculated by subtracting non-specific from total counts determined in a beta counter and reflected binding to MOR expressed on the cell surface. Specific counts were compared between sucrose pre-treated (maximum binding) and untreated cells using the paired t-test. Lower CPM in the untreated cells was interpreted as receptor internalization. Using GraphPad Prism 4.0c, values were then plotted as percentages of maximum binding.

### 3'-5'-cyclic adenosine monophosphate enzyme-immunoassay (cAMP EIA)

Opioid-induced inhibition of cAMP formation was assessed using the cAMP Biotrak Enzymeimmunoassay (GE Healthcare). HEK293 cells were seeded in 96-well plates and transfected with rat/NMR pCMV-*oprm1*-IRES-eGFP vectors for 18-36 h prior to the assay. Cells were washed twice with PBS and incubated for 20 min with 100 µl extracellular solution (ECS: 2 mM CaCl2, 10 mM Glucose, 10 mM HEPES, 5 mM KCl, 2 mM MgCl2, 140 mM NaCl, adjusted to pH 7.5 with NaOH) containing either 1) no drug, 2) the cAMP activator forskolin (10 µM) + the phosphodiesterase inhibitor IBMX (2 µM) (FI), 3) FI + the MOR agonist DAMGO (10 µM), or 4) FI + DAMGO + NLX (20 µM). The ECS was then removed and cells were lysed for 10 min at room temperature with 200 µl lysis reagent. Next, 100 µl of lysate was transferred to an antigen-coated (donkey anti-rabbit IgG) EIA plate. All samples were arranged in duplicates. cAMP levels were detected with a microplate reader at 450 nm using SOFTmax® Pro software. All cAMP values were normalized to total amount of protein measured in each sample immediately following lysis using the Bradford method. Values were statistically analyzed using the Friedman test followed by Dunn's Multiple Comparison Test to compare the effect of DAMGO or DAMGO plus NLX on the formation of cAMP following forskolin treatment for each vector. To compare the effects between the rat and NMR MOR, data were transformed into % of forskolin-induced cAMP levels and analyzed using Kruskal-Wallis test followed by Dunn's Multiple Comparison Test. 

### Imaging of cellular MOR localization

HEK293 were plated on 1.2 cm polylysine-coated glass slides and transiently transfected with either WT rat or NMR pCMV-*oprm1-*eGFP vector DNA one day prior to imaging. Cells were washed three times in PBS and incubated with either PBS, 10 µM DAMGO in PBS, or 10 µM morphine-sulfate in PBS for 20-30 min at 37°C. Cells were then washed again in PBS and fixed for 15 min in 4 % PFA and 4% sucrose. After blocking (30 min in PBS containing 5% normal goat serum and 0.3% Triton™ X-100) slides were incubated overnight at 4°C with polyclonal rabbit anti-EEA1 (early endosomal marker 1) (Cell Signaling Technologies, 1:1000 in PBS containing 1% BSA and 0.3% Triton™ X-100). Slides were washed three times using PBS and incubated for 1 h at room temperature with the secondary antibody (Alexa Fluor® 568 Goat Anti-Rabbit IgG, Life Technologies, 1:200 in PBS containing 1% BSA and 0.3% Triton™ X-100). After washing in PBS, the glass slides were mounted on microscope slides with Mowiol® 4-88. Slides were dried overnight and viewed under a Zeiss LSM 510 Meta confocal laser scanning microscope. Fluorescence images were obtained using excitation wavelengths of 543 nm and 488 nm (63 x oil-immersion objective). Z-stacks were performed on 3.8-4.1 x zoomed regions usually showing 2-6 transfected cells. Every 0.38 µM a picture was taken and the pinhole size was set to 0.7 µM. Co-localization of EEA1 and eGFP was analyzed in vesicular structures using ImageJ 1.46r (Wayne Rasband, National Institutes of Health) software and the ImageJ plugin Organelle Based Colocalisation (OBCOL) 1.45 (Nick Hamilton, Ben Woodcroft, Luke Hammond, and Jenny Stow, Institute for Molecular Bioscience, The University of Queensland, Queensland, Australia). All unwanted content (e.g. non-transfected cells, improper stack slices) was removed from the stacks, and then the channels were split. A lower intensity threshold was set for each channel (usually 35 for eGFP and 65 for EEA1). The two converted single channel stacks were then added to OBCOL. After processing the co-localization and object, statistics for each individual object was performed setting the minimal pixel filter to 250-10,000 pixels. The Manders’ coefficients representing the % overlap of eGFP and EEA1 (M1) of individual objects are given. 

## Results and Discussion

### Sequencing and analysis of the NMR *oprm1*

We first sequenced and analyzed the complete CDS of the NMR *oprm1*. Primers were designed based on the most broadly conserved regions of the mammalian *oprm1* (Table S1). Segments ranging from 250-700 base pairs were produced using PCR amplification of NMR gDNA and cDNA and assembled into a single sequence by aligning overlapping regions. The CDS is 1,203 nucleotides long and codes for a 400 aa long 7-transmembrane G-protein coupled receptor (GPCR) highly similar (greater than 90% nucleotide match) to *oprm1* variants comprising exons 1, 2, 3 and 4 of a range of species including humans, rats and mice (Figure 1A). While conducting the experiments, Kim et al. sequenced the whole NMR genome. Our NMR *oprm1* CDS had a nearly 100% match to the 4 sequential exons published [25]. Moreover, recent transcriptome sequencing of the NMR by Szafranski and colleagues revealed a perfect match with our NMR *oprm1* CDS [26]. Three nucleotide substitution sites were identified in highly conserved regions of the 5’-end of the *oprm1* resulting in three unique aa at the N-terminal NMR MOR compared to all other species examined (Figure 1B).

### [H3]DAMGO binding is stronger in WT rat compared to NMR MOR

**Figure 2:**
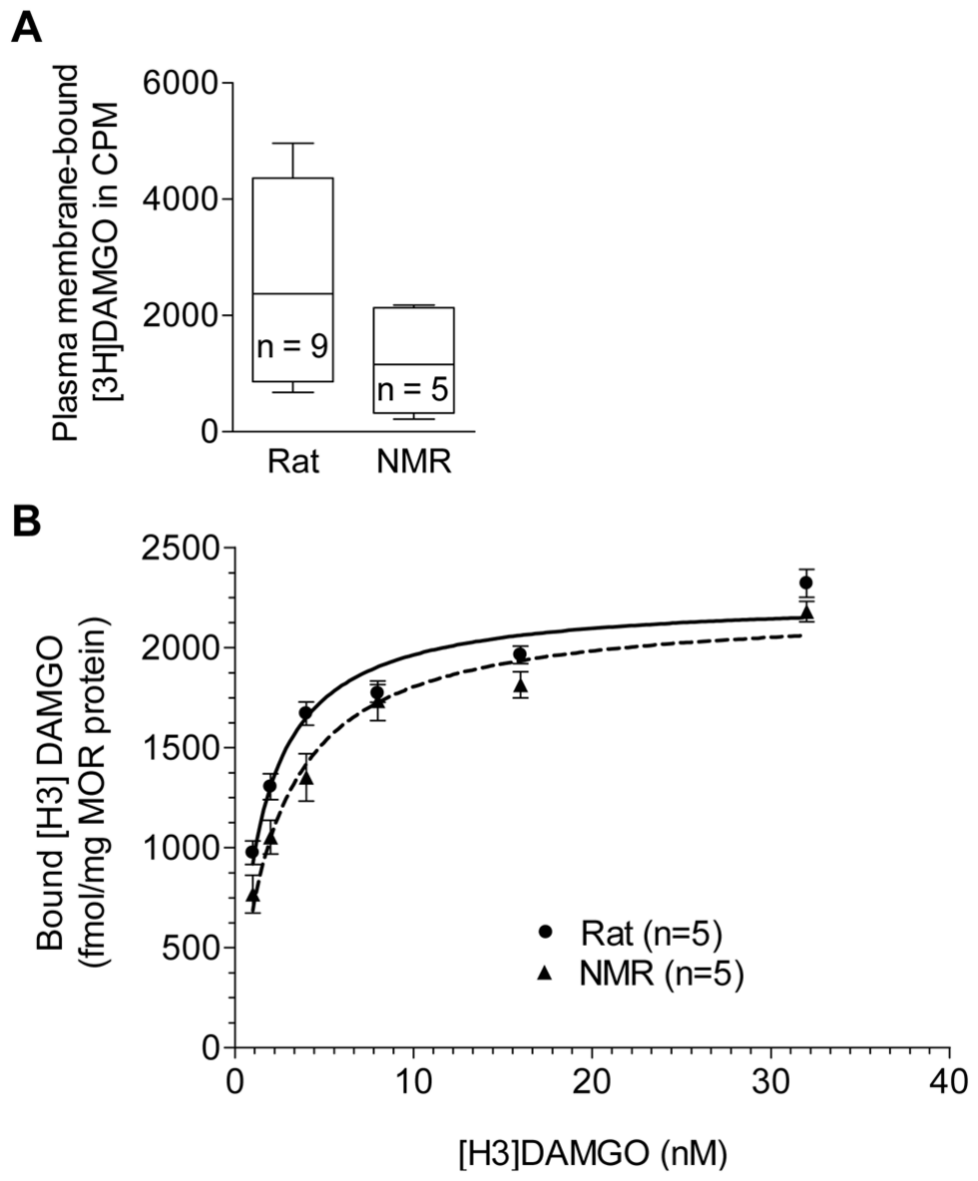
Radioligand binding of rat and NMR MOR. **A**) [H3]DAMGO binding to plasma membrane extracts of rat and NMR MOR expressed in HEK293 cells (pCMV-*oprm1*-IRES-eGFP vector DNAs). Data are shown as whisker blots (min to max). **B**) Normalized [H3]DAMGO saturation binding curve of rat and NMR MOR expressed in HEK293 cells (pCMV-*oprm1*-IRES-eGFP vector DNAs) using whole membrane preparations. Data represent means ± SEM. Statistical analysis was performed using the two-tailed Mann Whitney U test.

Upon completion of the NMR and rat pCMV-*oprm1*-IRES-eGFP vectors, we checked their ability to bind opioid ligands. Plasma membrane extracts of transiently transfected HEK293 cells were analyzed using radioligand binding assays with the tritium-tagged MOR-selective agonist DAMGO. Both receptors bound [3H]DAMGO showing that MOR was expressed and integrated into the cell membrane (Figure 2A). No significant difference between rat and NMR MOR was detected in CPM values (two-tailed Mann Whitney U test, p > 0.05). To account for variations in the amount of transiently expressed receptor, the amount of bound ligand per fmol of MOR was determined in saturation binding assays. Data nomalized to the amount of MOR expressed were then calculated as area under the curve (AUC) of the non-linear regression fit according to one-site binding. In membrane preparations of transiently transfected HEK293 cells, the NMR MOR bound significantly lower amounts of [H3]DAMGO (AUC: 55,631 ± 1,675) compared to the rat MOR (AUC: 60,218 ± 482.3; two-tailed Mann Whitney U test, p < 0.05), indicating that the NMR receptor bound [H3]DAMGO with less affinity compared to WT rat MOR (Figure 2B). Consistently, the dissociation constant (Kd) of [H3]DAMGO and MOR was significantly higher for the NMR (2.42 ± 0.48 nM) as compared to the rat (1.45 ± 0.11 nM) (two-tailed Mann Whitney U test, p < 0.05). These results may provide a molecular explanation for the finding that NMR require higher doses of opioids to induce analgesia *in vivo* [27,28]. Our data resemble an *in vitro* saturation binding study showing lower binding of DAMGO to the human MOR variant D40 as compared to the WT N40 [19]. Consistently, a reduced *in vivo* MOR receptor binding potential of D40 was described [16,17] and patients homozygous for D40 showed increased post-operative morphine demands [6–9]. There is some evidence that the reduced surface expression of D40 is the consequence of the loss of one out of five glycosylation sites in the human MOR [19,29]. Due to a glycine instead of serine at aa 11, the NMR MOR contains only three putative glycosylation sites but the receptor density and expression level in NMR tissues is unknown so far. 

### MOR-mediated cAMP inhibition is similar in rat and NMR

**Figure 3:**
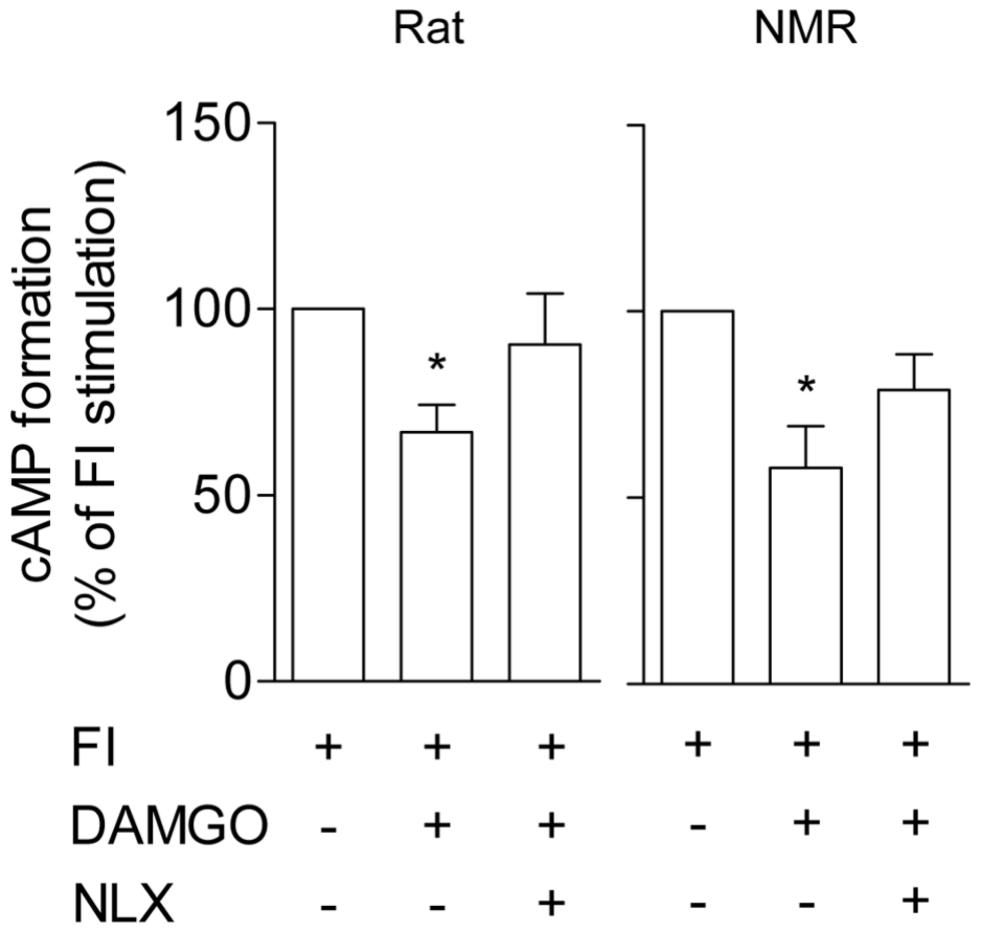
cAMP inhibition by rat and NMR MOR activation. Intracellular cAMP levels were measured using EIA in HEK293 cells transfected with NMR (n = 4) and rat (n = 6) pCMV-*oprm1*-IRES-eGFP vector DNAs following treatment with forskolin/IBMX (FI; 10 µM/2 µM) and either DAMGO (10 µM) or DAMGO and NLX (20 µM). Data are presented as the percentage of FI-stimulated cAMP formation (means ± SEM). Statistical analysis was performed on raw data (fmol) after normalization to protein. Friedman test and Dunn's Multiple Comparison Test, * p < 0.05 compared to FI-stimulated cAMP formation.

Next we examined differences in downstream signaling. In general, opioid binding activates inhibitory Gα subunits, thereby reducing adenylyl cyclase activity which decreases cAMP formation [30]. Forskolin-induced cAMP formation was significantly reduced following DAMGO application in both rat (161 ± 59 fmol/1 µg protein in FI *versus* 100 ± 32 fmol/1 µg protein in FI + DAMGO, Friedman test and Dunn's Multiple Comparison Test, p < 0.05) and NMR MOR (213 ± 54 fmol/1 µg protein in FI *versus* 136 ± 40 fmol/1 µg protein in FI + DAMGO, Friedman test and Dunn's Multiple Comparison Test, p < 0.05) (see also Figure 3). This inhibition of forskolin-induced cAMP formation was not significantly different between the NMR (-42 ± 11%) and rat MOR (-33 ± 7%) (Mann Whitney U test, p > 0.05) and naloxone reduced the DAMGO effect (Figure 3), in accordance with other findings [31]. Previous *in vitro* analysis of MOR mutants revealed no significant changes in morphine-induced cAMP inhibition at high (1 µM) [18] but demonstrated differences at low (1-100 nM) agonist concentrations [19]. It is possible that no differences in cAMP inhibition were detected in the present study due to the high dose of DAMGO used (10 µM). 

### Rat and NMR MOR show similar trafficking in response to DAMGO and morphine

**Figure 4:**
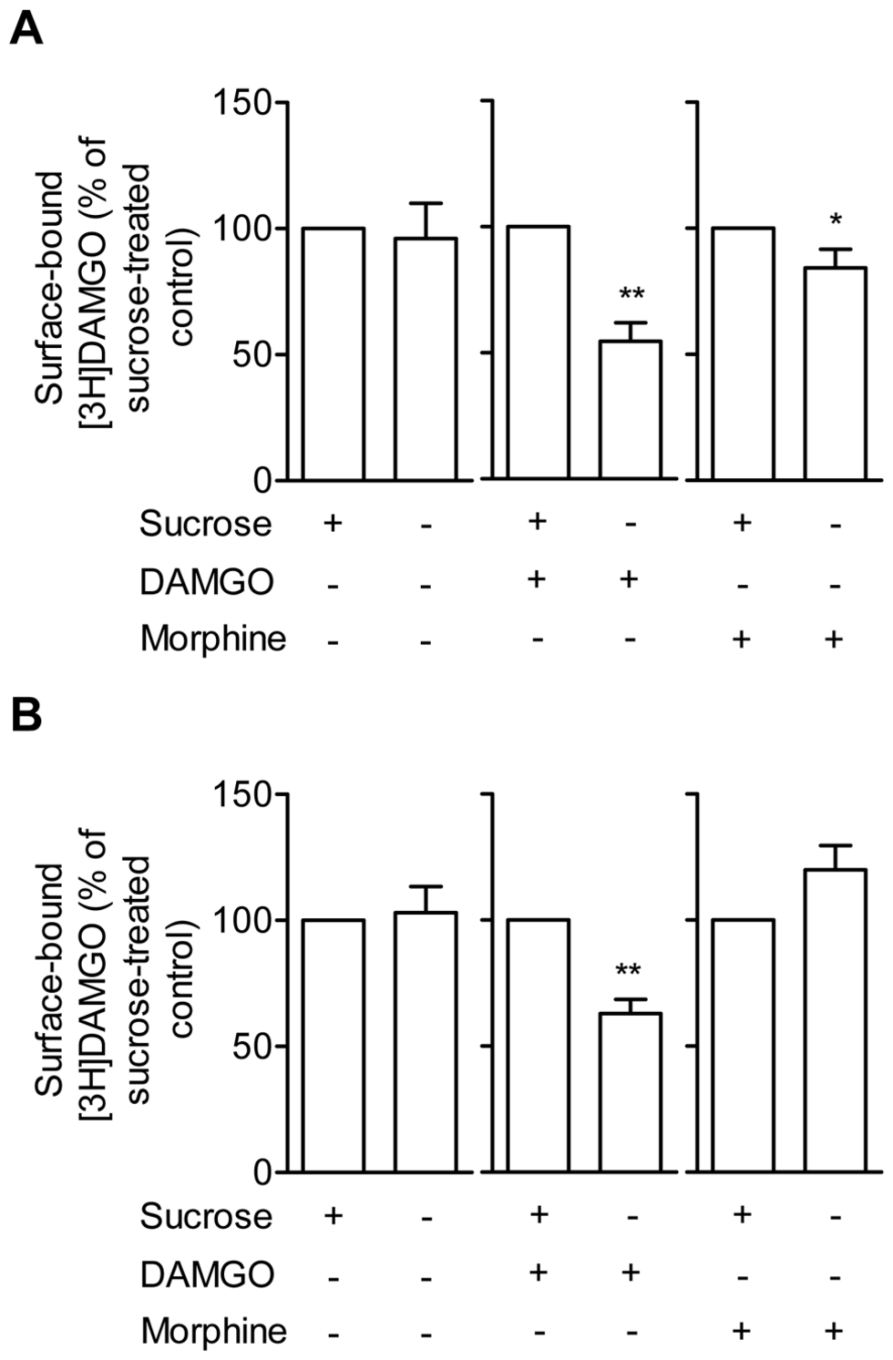
Surface expression of rat and NMR MOR following agonist stimulation. HEK293 cells transiently transfected with either **A**) rat or **B**) NMR pCMV-*oprm1*-IRES-eGFP vector DNA were incubated with either HBSS buffer (control), 10 µM DAMGO, or 10 µM morphine for 30 min at 37°C. To block internalization, cells were pre-treated with sucrose. Removal of bound but not internalized ligand was performed in low pH stripping buffer before cells were exposed to [3H]DAMGO. Six independent experiments were performed. Data represent surface-bound CPM in % of sucrose pre-treated cells (means ± SEM). Statistical analysis was performed using the paired t-test, * p < 0.05 and ** p < 0.01.

Finally we determined agonist-induced MOR trafficking by whole cell radioligand binding of the rat and NMR MOR. In agreement with previous literature [32,33], DAMGO (but not morphine or vehicle) markedly reduced surface-bound radioligand without sucrose pretreatment in rat MOR, suggesting endocytosis (paired t-test, p < 0.01, Figure 4A). While no significant CPM effect of sucrose pretreatment was found in vehicle-treated cells (paired t-test, p > 0.05, Figure 4A), some internalization was observed in three out of six experiments. This may reflect constitutive receptor recycling. Also, the NMR MOR showed reduced surface binding in response to DAMGO (paired t-test, p < 0.001, Figure 4B) but not to morphine or vehicle (paired t-test, p > 0.05, Figure 4B). The percentage of sucrose-blocked endocytosis did not differ between the WT rat and NMR MOR (unpaired t-test, p > 0.05), suggesting similar receptor trafficking in the two species. 

To confirm these findings we used confocal microscopy of cells transfected with pCMV-NMR*oprm1-*eGFP. Opioid receptor fusions with eGFP have previously been shown to have no effect on receptor binding, signaling, or trafficking when expressed in HEK293 cells [34,35]. Plasma membrane extracts of transiently transfected HEK293 cells expressing eGFP-tagged MOR were analyzed using radioligand binding to verify surface expression. The receptor was expressed and integrated into the cell membrane (Figure S1A). Cells were stained for EEA1 to differentiate between eGFP-positive vesicular structures trafficking towards the cell membrane and eGFP-positive endosomes. The NMR MOReGFP showed a reasonable increase in the number of vesicular objects with high eGFP/EEA1 co-expression in DAMGO-treated cells in comparison to untreated control cells, while only few of such double-positive objects were observed in morphine-treated cells. Sequencing of the plasmid verified that pCMV-NMR*oprm1*eGFP was intact. These findings indicate internalization of the NMR MOReGFP predominantly after DAMGO and to a lesser degree after morphine stimulation, which is similar to the findings of others [33,36,37] and supports our data obtained in the whole cell ligand binding assay. 

## Conclusions

Comparison of the NMR MOR sequence to other mammals revealed high overall homology but also highlighted some unique aa. We found differences in DAMGO binding between the NMR and rat MOR, while cAMP inhibition was similar. In addition, both receptors internalized strongly after DAMGO but less so after morphine stimulation. Taken together, we conclude that the NMR MOR is functionally indifferent from the rat receptor.

How do these findings compare to the unusual NMR behavior in response to morphine? Reduced stepping latencies after opioid application in the hot-plate test [4,38,39] may have been due to the known hyperlocomotion induced by high doses of morphine [40,41] since opioids demonstrated normal analgesic action in the NMR in another pain test (formalin-induced pain) [5,27,28]. Together with the data presented here we suggest that the NMR MOR displays normal functionality in terms of locomotion and pain inhibition. 

A remaining question is how MOR agonists induce aggression in the NMR. In animals treated with ≥ 10 mg/kg morphine or ≥ 20 mg/kg pethidine aggression was so strong that all animal housed together died due to vigorous fighting [4,38]. The present study did not provide an explanation of these findings. To address this issue examination of MOR density and localization in the NMR nervous system may be necessary. In this context, an altered connectivity of TRPV1 channels was found to be responsible for the NMR’s behavioral insensitivity to capsaicin [42] and polymorphism-dependent differential localization and density of the A112 and G112 SNPs of the mouse MOR were observed throughout the mouse brain [43]. The NMR’s extreme reaction to opioids surely makes it difficult to investigate opioid analgesia but studying the relationship between the anatomical localization of opioid receptors and the behavioral outcomes may promise new insights. 

## Supporting Information

Figure S1 **Internalization of NMR MOR-eGFP following agonist stimulation.** HEK293 cells were transiently transfected with NMR pCMV-*oprm1*eGFP vector DNA to express MOR with a C-terminal eGFP tag for confocal analysis. A) Surface expression of NMR MOReGFP fusion protein was analyzed using radioligand binding to plasma membrane extracts. Data are shown as whisker blots (min to max, n = 5). **B**) Cells were incubated with either PBS (control), 10 µM DAMGO, or 10 µM morphine. PFA-fixed cells were then stained for EEA1, mounted in Mowiol and imaged with a Zeiss LSM 510 Meta confocal laser scanning microscope. Z-stacks were analyzed as detailed in the methods. Data represent Manders’ coefficients (M1, overlap of MOReGFP to EEA1; 1 = 100% co-localization, 0 = no co-localization) determined in vesicular objects across 2-3 stacks per slide. Three slides were analyzed per treatment.
(TIF)

Table S1 **Primers used to sequence the NMR *oprm1*.**
(DOC)

Table S2 **Transfection primers used to clone the complete CDS of NMR and rat *oprm1* into empty pIRES2-eGFP vector.**
(DOC)

Table S3 **Mutagenesis primer used to excise IRES from NMR pCMV-*oprm1*-IRES-eGFP.**
(DOC)

Text S1 **Rat pCMV-*oprm1*-IRES-eGFP Vector map.**
(PDF)

Text S2 **NMR pCMV-*oprm1*-IRES-eGFP Vector map.**
(PDF)

Text S3 **NMR pCMV-*oprm1*eGFP Vector map.**
(PDF)
