# Correction: Functional Characteristics of the Naked Mole Rat μ-Opioid Receptor

**DOI:** 10.1371/annotation/d86135c4-7a99-43fb-a3ea-445a9e671c51

**Published:** 2014-01-06

**Authors:** Melanie Busch-Dienstfertig, Clarisse A. Roth, Christoph Stein

The equal contributions designation was incorrectly assigned. Clarisse A. Roth and Melanie Busch-Dienstfertig should be noted as having contributed equally to this work.

