# Supplementary figures and images for: Functional Characteristics of the Naked Mole Rat μ-Opioid Receptor
Source: PLoS One. 2013 Nov 27;8(11):e79121. doi: 10.1371/journal.pone.0079121 (PMC3842265; doi:10.1371/journal.pone.0079121)

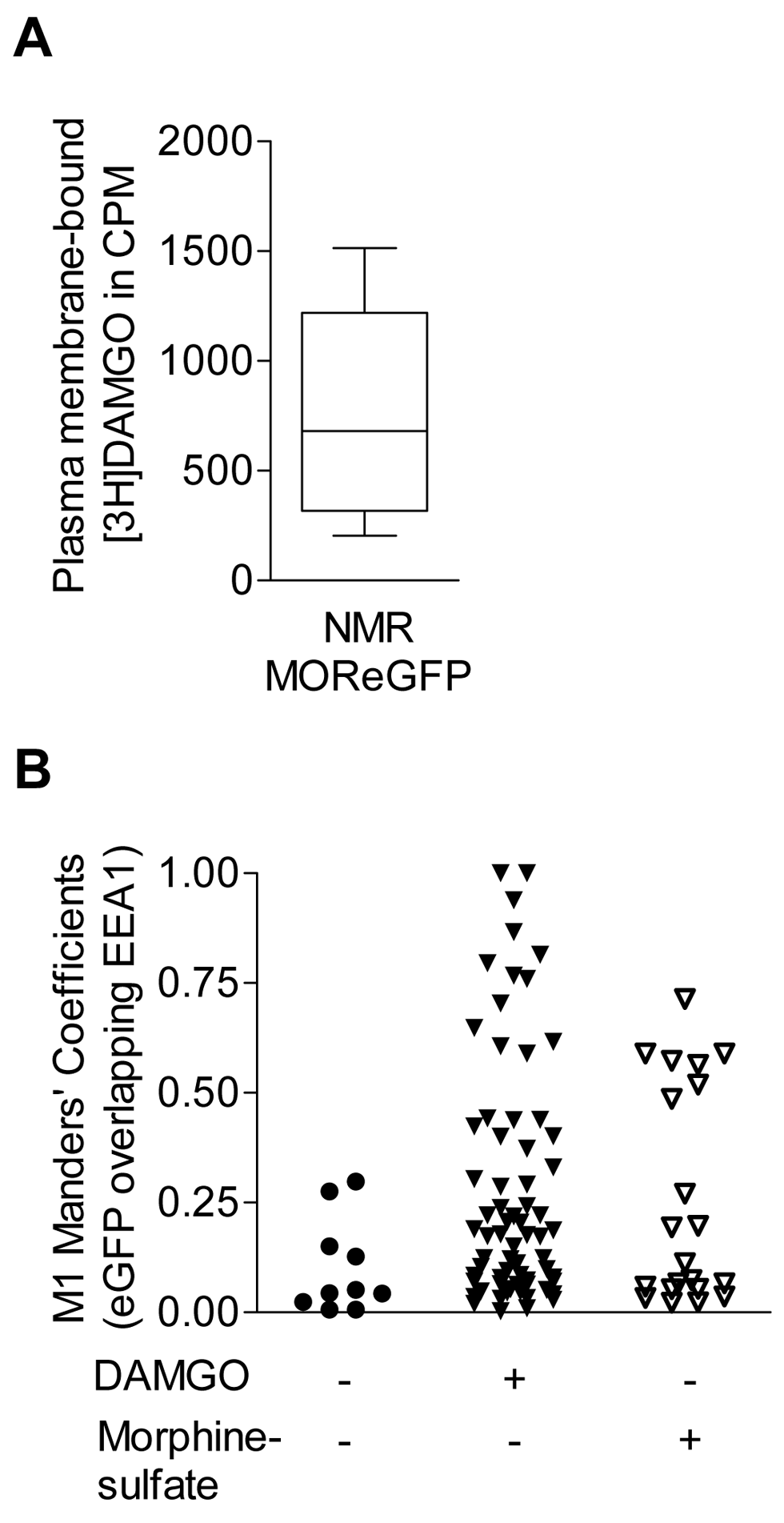

Supplement: Figure S1 — Internalization of NMR MOR-eGFP following agonist stimulation. HEK293 cells were transiently transfected with NMR pCMV-oprm1eGFP vector DNA to express MOR with a C-terminal eGFP tag for confocal analysis. A) Surface expression of NMR MOReGFP fusion protein was analyzed using radioligand binding to plasma membrane extracts. Data are shown as whisker blots (min to max, n = 5). B) Cells were incubated with either PBS (control), 10 µM DAMGO, or 10 µM morphine. PFA-fixed cells were then stained for EEA1, mounted in Mowiol and imaged with a Zeiss LSM 510 Meta confocal laser scanning microscope. Z-stacks were analyzed as detailed in the methods. Data represent Manders’ coefficients (M1, overlap of MOReGFP to EEA1; 1 = 100% co-localization, 0 = no co-localization) determined in vesicular objects across 2-3 stacks per slide. Three slides were analyzed per treatment. (TIF) [file pone.0079121.s001.tif]
