# Supplementary material for: Functional Characteristics of the Naked Mole Rat μ-Opioid Receptor
Source: PLoS One. 2013 Nov 27;8(11):e79121. doi: 10.1371/journal.pone.0079121 (PMC3842265; doi:10.1371/journal.pone.0079121)
Supplement: Table S1 — Primers used to sequence the NMR oprm1. (DOC) [file pone.0079121.s002.doc]

| **Primer Name** | **Sequence (5’** → **3’)** |
| --- | --- |
| Cav3’A | ATTCAGACCCTCAGTGAGAC |
| Cav3’B | AGACAGCTCCTTGCCCTAAC |
| Cav3’C | TCTCACACCATTCAGACCCTC |
| MOP2B | GTAATGTTCATGGCAACCACAAAATACAGG |
| MOP1A-FW | AAGCGGCTGAGGCGC |
| MOP1B-RV | GACCGGCAGCCC |
| NMRex1asB | TCCTGGTCATGTATGTGATC |
