# Supplementary material for: Functional Characteristics of the Naked Mole Rat μ-Opioid Receptor
Source: PLoS One. 2013 Nov 27;8(11):e79121. doi: 10.1371/journal.pone.0079121 (PMC3842265; doi:10.1371/journal.pone.0079121)
Supplement: Table S2 — Transfection primers used to clone the complete CDS of NMR and rat oprm1 into empty pIRES2-eGFP vector. (DOC) [file pone.0079121.s003.doc]

| **Primer Name** | **Sequence (5’** → **3’)** | **Restriction Enzyme** |
| --- | --- | --- |
| Rat-F | ActcgagAAGGACAGCAGCACCGGCCCAGGG | XhoI |
| Rat-R | GCTCCATTGCCCTAAgaattcTGCA | PstI |
| NMR-F | AGctcgagAGCACCATGGACAGCAGTGT | EcoRI |
| NMR-R | GTTGCCCTAACTGGGTCTTGctgcagATGTA | SacI |

*Lower case letters indicate restriction enzyme binding site
