# Supplementary material for: Functional Characteristics of the Naked Mole Rat μ-Opioid Receptor
Source: PLoS One. 2013 Nov 27;8(11):e79121. doi: 10.1371/journal.pone.0079121 (PMC3842265; doi:10.1371/journal.pone.0079121)
Supplement: Table S3 — Mutagenesis primer used to excise IRES from NMR pCMV-oprm1-IRES-eGFP. (DOC) [file pone.0079121.s004.doc]

| **Primer Name** | **Sequence (5’** → **3’)** |
| --- | --- |
| NMR delete IRES FW | CAgCTCCgTTgCCCATggTgAgCAAggg |
| NMR delete IRES RV | CCCTTgCTCACCATgggCAACggAgCTg |
