# Supplementary material for: Functional Characteristics of the Naked Mole Rat μ-Opioid Receptor
Source: PLoS One. 2013 Nov 27;8(11):e79121. doi: 10.1371/journal.pone.0079121 (PMC3842265; doi:10.1371/journal.pone.0079121)
Supplement: Text S1 — Rat pCMV-oprm1-IRES-eGFP Vector map. (PDF) [file pone.0079121.s005.pdf]

Rat pCMV-*oprm1*-IRES-eGFP Vector map

LOCUS pCMV-Rat *oprm1*-IRES-eGFP 6498 bp ds-DNA circular
ORGANISM Rat (*Rattus norvegicus*)
FEATURES Location/Qualifiers
CMV-Promotor 10..589
Rat *oprm1* 622..1818
IRES 1856..2440
eGFP 2444..3163
SV40_promoter 4175..4354
Kan/neoR 4394..5188

ORIGIN
1 TAGTTATTAA TAGTAATCAA TTACGGGGTC ATTAGTTCAT AGCCCATATA TGGAGTTCCG
61 CGTTACATAA CTTACGGTAA ATGGCCCGCC TGGCTGACCG CCCAACGACC CCCGCCCATT
121 GACGTCAATA ATGACGTATG TTCCCATAGT AACGCCAATA GGGACTTTCC ATTGACGTCA
181 ATGGGTGGAG TATTTACGGT AAACTGCCCA CTTGGCAGTA CATCAAGTGT ATCATATGCC
241 AAGTACGCCC CCTATTGACG TCAATGACGG TAAATGGCCC GCCTGGCATT ATGCCCAGTA
301 CATGACCTTA TGGGACTTTC CTACTTGGCA GTACATCTAC GTATTAGTCA TCGCTATTAC
361 CATGGTGATG CGGTTTTGGC AGTACATCAA TGGGCGTGGA TAGCGGTTTG ACTCACGGGG
421 ATTTCCAAGT CTCCACCCCA TTGACGTCAA TGGGAGTTTG TTTTGGCACC AAAATCAACG
481 GGACTTTCCA AAATGTCGTA ACAACTCCGC CCCATTGACG CAAATGGGCG GTAGGCGTGT
541 ACGGTGGGAG GTCTATATAA GCAGAGCTGG TTTAGTGAAC CGTCAGATCC GCTAGCGCTA
601 CCGGACTCAG ATCTCGAGCT C**ATG**GACAGC AGCACCGGCC CAGGGAACAC CAGCGACTGC
661 TCAGACCCCT TAGCTCAGGC AAGTTGCTCC CCAGCACCTG GCTCCTGGCT CAACTTGTCC
721 CACGTTGATG GCAACCAGTC CGATCCATGC GGTCTGAACC GCACCGGGCT TGGCGGGAAC
781 GACAGCCTGT GCCCTCAGAC CGGCAGCCCT TCCATGGTCA CAGCCATTAC CATCATGGCC
841 CTCTACTCTA TCGTGTGTGT AGTGGGCCTC TTCGGAAACT TCCTGGTCAT GTATGTGATT
901 GTAAGATACA CCAAAATGAA GACTGCCACC AACATCTACA TTTTCAACCT TGCTCTGGCA
961 GACGCCTTAG CGACCAGTAC ACTGCCCTTT CAGAGTGTCA ACTACCTGAT GGGAACATGG
1021 CCCTTCGGAA CCATCCTCTG CAAGATCGTG ATCTCAATAG ATTACTACAA CATGTTCACC
1081 AGCATATTCA CCCTCTGCAC CATGAGCGTG GACCGCTACA TTGCTGTCTG CCACCCAGTC
1141 AAAGCCCTGG ATTTCCGTAC CCCCCGAAAT GCCAAAATCG TCAACGTCTG CAACTGGATC
1201 CTCTCTTCTG CCATCGGTCT GCCTGTAATG TTCATGGCAA CCACAAAATA CAGGCAGGGG
1261 TCCATAGATT GCACCCTCAC GTTCTCCCAC CCAACCTGGT ACTGGGAGAA CCTGCTCAAA
1321 ATCTGTGTCT TTATCTTCGC TTTCATCATG CCGGTCCTCA TCATCACTGT GTGTTACGGC
1381 CTGATGATCT TACGACTCAA GAGCGTTCGC ATGCTATCGG GCTCCAAAGA AAAGGACAGG
1441 AATCTGCGCA GGATCACCCG GATGGTGCTG GTGGTCGTGG CTGTATTTAT CGTCTGCTGG
1501 ACCCCCATCC ACATCTACGT CATCATCAAA GCGCTGATCA CGATTCCAGA AACCACATTT
1561 CAGACCGTTT CCTGGCACTT CTGCATTGCT TTGGGTTACA CGAACAGCTG CCTGAATCCA
1621 GTTCTTTACG CCTTCCTGGA TGAAAACTTC AAGCGATGCT TCAGAGAGTT CTGCATCCCA
1681 ACCTCGTCCA CGATCGAACA GCAAAACTCC ACTCGAGTCC GTCAGAACAC TAGGGAACAT
1741 CCCTCCACGG CTAATACAGT GGATCGAACT AACCACCAGC TAGAAAATCT GGAGGCAGAA
1801 ACTGCTCCAT TGCCC**TAA**GA ATTCTGCAGT CGACGGTACC GCGGGCCCGG GATCC*GCCCC*
1861 *TCTCCCTCCC CCCCCCCTAA CGTTACTGGC CGAAGCCGCT TGGAATAAGG CCGGTGTGCG*
1921 *TTTGTCTATA TGTTATTTTC CACCATATTG CCGTCTTTTG GCAATGTGAG GGCCCGGAAA*
1981 *CCTGGCCCTG TCTTCTTGAC GAGCATTCCT AGGGGTCTTT CCCCTCTCGC CAAAGGAATG*
2041 *CAAGGTCTGT TGAATGTCGT GAAGGAAGCA GTTCCTCTGG AAGCTTCTTG AAGACAAACA*
2101 *ACGTCTGTAG CGACCCTTTG CAGGCAGCGG AACCCCCCAC CTGGCGACAG GTGCCTCTGC*
2161 *GGCCAAAAGC CACGTGTATA AGATACACCT GCAAAGGCGG CACAACCCCA GTGCCACGTT*
2221 *GTGAGTTGGA TAGTTGTGGA AAGAGTCAAA TGGCTCTCCT CAAGCGTATT CAACAAGGGG*
2281 *CTGAAGGATG CCCAGAAGGT ACCCCATTGT ATGGGATCTG ATCTGGGGCC TCGGTGCACA*
2341 *TGCTTTACAT GTGTTTAGTC GAGGTTAAAA AAACGTCTAG GCCCCCCGAA CCACGGGGAC*
2401 *GTGGTTTTCC TTTGAAAAAC ACGATGATAA TATGGCCACA* ACC**ATG**GTGA GCAAGGGCGA
2461 GGAGCTGTTC ACCGGGGTGG TGCCCATCCT GGTCGAGCTG GACGGCGACG TAAACGGCCA
2521 CAAGTTCAGC GTGTCCGGCG AGGGCGAGGG CGATGCCACC TACGGCAAGC TGACCCTGAA
2581 GTTCATCTGC ACCACCGGCA AGCTGCCCGT GCCCTGGCCC ACCCTCGTGA CCACCCTGAC
2641 CTACGGCGTG CAGTGCTTCA GCCGCTACCC CGACCACATG AAGCAGCACG ACTTCTTCAA
2701 GTCCGCCATG CCCGAAGGCT ACGTCCAGGA GCGCACCATC TTCTTCAAGG ACGACGGCAA
2761 CTACAAGACC CGCGCCGAGG TGAAGTTCGA GGGCGACACC CTGGTGAACC GCATCGAGCT
2821 GAAGGGCATC GACTTCAAGG AGGACGGCAA CATCCTGGGG CACAAGCTGG AGTACAACTA
2881 CAACAGCCAC AACGTCTATA TCATGGCCGA CAAGCAGAAG AACGGCATCA AGGTGAACTT
2941 CAAGATCCGC CACAACATCG AGGACGGCAG CGTGCAGCTC GCCGACCACT ACCAGCAGAA
3001 CACCCCCATC GGCGACGGCC CCGTGCTGCT GCCCGACAAC CACTACCTGA GCACCCAGTC
3061 CGCCCTGAGC AAAGACCCCA ACGAGAAGCG CGATCACATG GTCCTGCTGG AGTTCGTGAC
3121 CGCCGCCGGG ATCACTCTCG GCATGGACGA GCTGTACAAG **TAA**AGCGGCC GCGACTCTAG

```
3181 ATCATAATCA GCCATACCAC ATTTGTAGAG GTTTTACTTG CTTTAAAAAA CCTCCCACAC
3241 CTCCCCCTGA ACCTGAAACA TAAAATGAAT GCAATTGTTG TTGTTAACTT GTTTATTGCA
3301 GCTTATAATG GTTACAAATA AAGCAATAGC ATCACAAATT TCACAAATAA AGCATTTTTT
3361 TCACTGCATT CTAGTTGTGG TTTGTCCAAA CTCATCAATG TATCTTAAGG CGTAAATTGT
3421 AAGCGTTAAT ATTTTGTTAA AATTCGCGTT AAATTTTTGT TAAATCAGCT CATTTTTTAA
3481 CCAATAGGCC GAAATCGGCA AAATCCCTTA TAAATCAAAA GAATAGACCG AGATAGGGTT
3541 GAGTGTTGTT CCAGTTTGGA ACAAGAGTCC ACTATTAAAG AACGTGGACT CCAACGTCAA
3601 AGGGCGAAAA ACCGTCTATC AGGGCGATGG CCCACTACGT GAACCATCAC CCTAATCAAG
3661 TTTTTTGGGG TCGAGGTGCC GTAAAGCACT AAATCGGAAC CCTAAAGGGA GCCCCCGATT
3721 TAGAGCTTGA CGGGGAAAGC CGGCGAACGT GGCGAGAAAG GAAGGGAAGA AAGCGAAAGG
3781 AGCGGGCGCT AGGGCGCTGG CAAGTGTAGC GGTCACGCTG CGCGTAACCA CCACACCCGC
3841 CGCGCTTAAT GCGCCGCTAC AGGGCGCGTC AGGTGGCACT TTTCGGGGAA ATGTGCGCGG
3901 AACCCCTATT TGTTTATTTT TCTAAATACA TTCAAATATG TATCCGCTCA TGAGACAATA
3961 ACCCTGATAA ATGCTTCAAT AATATTGAAA AAGGAAGAGT CCTGAGGCGG AAAGAACCAG
4021 CTGTGGAATG TGTGTCAGTT AGGGTGTGGA AAGTCCCCAG GCTCCCCAGC AGGCAGAAGT
4081 ATGCAAAGCA TGCATCTCAA TTAGTCAGCA ACCAGGTGTG GAAAGTCCCC AGGCTCCCCA
4141 GCAGGCAGAA GTATGCAAAG CATGCATCTC AATTAGTCAG CAACCATAGT CCCGCCCCTA
4201 ACTCCGCCCA TCCCGCCCCT AACTCCGCCC AGTTCCGCCC ATTCTCCGCC CCATGGCTGA
4261 CTAATTTTTT TTATTTATGC AGAGGCCGAG GCCGCCTCGG CCTCTGAGCT ATTCCAGAAG
4321 TAGTGAGGAG GCTTTTTTGG AGGCCTAGGC TTTTGCAAAG ATCGATCAAG AGACAGGATG
4381 AGGATCGTTT CGCATGATTG AACAAGATGG ATTGCACGCA GGTTCTCCGG CCGCTTGGGT
4441 GGAGAGGCTA TTCGGCTATG ACTGGGCACA ACAGACAATC GGCTGCTCTG ATGCCGCCGT
4501 GTTCCGGCTG TCAGCGCAGG GGCGCCCGGT TCTTTTTGTC AAGACCGACC TGTCCGGTGC
4561 CCTGAATGAA CTGCAAGACG AGGCAGCGCG GCTATCGTGG CTGGCCACGA CGGGCGTTCC
4621 TTGCGCAGCT GTGCTCGACG TTGTCACTGA AGCGGGAAGG GACTGGCTGC TATTGGGCGA
4681 AGTGCCGGGG CAGGATCTCC TGTCATCTCA CCTTGCTCCT GCCGAGAAAG TATCCATCAT
4741 GGCTGATGCA ATGCGGCGGC TGCATACGCT TGATCCGGCT ACCTGCCCAT TCGACCACCA
4801 AGCGAAACAT CGCATCGAGC GAGCACGTAC TCGGATGGAA GCCGGTCTTG TCGATCAGGA
4861 TGATCTGGAC GAAGAGCATC AGGGGCTCGC GCCAGCCGAA CTGTTCGCCA GGCTCAAGGC
4921 GAGCATGCCC GACGGCGAGG ATCTCGTCGT GACCCATGGC GATGCCTGCT TGCCGAATAT
4981 CATGGTGGAA AATGGCCGCT TTTCTGGATT CATCGACTGT GGCCGGCTGG GTGTGGCGGA
5041 CCGCTATCAG GACATAGCGT TGGCTACCCG TGATATTGCT GAAGAGCTTG GCGGCGAATG
5101 GGCTGACCGC TTCCTCGTGC TTTACGGTAT CGCCGCTCCC GATTCGCAGC GCATCGCCTT
5161 CTATCGCCTT CTTGACGAGT TCTTCTGAGC GGGACTCTGG GGTTCGAAAT GACCGACCAA
5221 GCGACGCCCA ACCTGCCATC ACGAGATTTC GATTCCACCG CCGCCTTCTA TGAAAGGTTG
5281 GGCTTCGGAA TCGTTTTCCG GGACGCCGGC TGGATGATCC TCCAGCGCGG GGATCTCATG
5341 CTGGAGTTCT TCGCCCACCC TAGGGGGAGG CTAACTGAAA CACGGAAGGA GACAATACCG
5401 GAAGGAACCC GCGCTATGAC GGCAATAAAA AGACAGAATA AAACGCACGG TGTTGGGTCG
5461 TTTGTTCATA AACGCGGGGT TCGGTCCCAG GGCTGGCACT CTGTCGATAC CCCACCGAGA
5521 CCCCATTGGG GCCAATACGC CCGCGTTTCT TCCTTTTCCC CACCCCACCC CCCAAGTTCG
5581 GGTGAAGGCC CAGGGCTCGC AGCCAACGTC GGGGCGGCAG GCCCTGCCAT AGCCTCAGGT
5641 TACTCATATA TACTTTAGAT TGATTTAAAA CTTCATTTTT AATTTAAAAG GATCTAGGTG
5701 AAGATCCTTT TTGATAATCT CATGACCAAA ATCCCTTAAC GTGAGTTTTC GTTCCACTGA
5761 GCGTCAGACC CCGTAGAAAA GATCAAAGGA TCTTCTTGAG ATCCTTTTTT TCTGCGCGTA
5821 ATCTGCTGCT TGCAAACAAA AAAACCACCG CTACCAGCGG TGGTTTGTTT GCCGGATCAA
5881 GAGCTACCAA CTCTTTTTCC GAAGGTAACT GGCTTCAGCA GAGCGCAGAT ACCAAATACT
5941 GTCCTTCTAG TGTAGCCGTA GTTAGGCCAC CACTTCAAGA ACTCTGTAGC ACCGCCTACA
6001 TACCTCGCTC TGCTAATCCT GTTACCAGTG GCTGCTGCCA GTGGCGATAA GTCGTGTCTT
6061 ACCGGGTTGG ACTCAAGACG ATAGTTACCG GATAAGGCGC AGCGGTCGGG CTGAACGGGG
6121 GGTTCGTGCA CACAGCCCAG CTTGGAGCGA ACGACCTACA CCGAACTGAG ATACCTACAG
6181 CGTGAGCTAT GAGAAAGCGC CACGCTTCCC GAAGGGAGAA AGGCGGACAG GTATCCGGTA
6241 AGCGGCAGGG TCGGAACAGG AGAGCGCACG AGGGAGCTTC CAGGGGGAAA CGCCTGGTAT
6301 CTTTATAGTC CTGTCGGGTT TCGCCACCTC TGACTTGAGC GTCGATTTTT GTGATGCTCG
6361 TCAGGGGGGC GGAGCCTATG GAAAAACGCC AGCAACGCGG CCTTTTTACG GTTCCTGGCC
6421 TTTTGCTGGC CTTTTGCTCA CATGTTCTTT CCTGCGTTAT CCCCTGATTC TGTGGATAAC
6481 CGTATTACCG CCATGCAT
```
//
