# Supplementary material for: Functional Characteristics of the Naked Mole Rat μ-Opioid Receptor
Source: PLoS One. 2013 Nov 27;8(11):e79121. doi: 10.1371/journal.pone.0079121 (PMC3842265; doi:10.1371/journal.pone.0079121)
Supplement: Text S2 — NMR pCMV-oprm1-IRES-eGFP Vector map. (PDF) [file pone.0079121.s006.pdf]

NMR pCMV-*oprm1*-IRES-eGFP Vector map

```
LOCUS       pCMV-NMR oprm1-IRES-eGFP        6512 bp ds-DNA   circular
ORGANISM  Naked mole rat (Heterocephalus glaber)
FEATURES             Location/Qualifiers
     CMV promoter    10..589
     NMR oprm1       625..1827
     IRES            1870..2454
     eGFP            2458..3177
     SV40_promoter   4189..4368
     Kan/neoR        4408..5202
ORIGIN
        1 TAGTTATTAA TAGTAATCAA TTACGGGGTC ATTAGTTCAT AGCCCATATA TGGAGTTCCG
       61 CGTTACATAA CTTACGGTAA ATGGCCCGCC TGGCTGACCG CCCAACGACC CCCGCCCATT
      121 GACGTCAATA ATGACGTATG TTCCCATAGT AACGCCAATA GGGACTTTCC ATTGACGTCA
      181 ATGGGTGGAG TATTTACGGT AAACTGCCCA CTTGGCAGTA CATCAAGTGT ATCATATGCC
      241 AAGTACGCCC CCTATTGACG TCAATGACGG TAAATGGCCC GCCTGGCATT ATGCCCAGTA
      301 CATGACCTTA TGGGACTTTC CTACTTGGCA GTACATCTAC GTATTAGTCA TCGCTATTAC
      361 CATGGTGATG CGGTTTTGGC AGTACATCAA TGGGCGTGGA TAGCGGTTTG ACTCACGGGG
      421 ATTTCCAAGT CTCCACCCCA TTGACGTCAA TGGGAGTTTG TTTTGGCACC AAAATCAACG
      481 GGACTTTCCA AAATGTCGTA ACAACTCCGC CCCATTGACG CAAATGGGCG GTAGGCGTGT
      541 ACGGTGGGAG GTCTATATAA GCAGAGCTGG TTTAGTGAAC CGTCAGATCC GCTAGCGCTA
      601 CCGGACTCAG ATCTCGAGAG CACCATGGAC AGCAGTGTCC TGCCCGGGAA CGCGGGCAAT
      661 TGCACCGATC CCTTCGCGCA ATCCAGTTGC TCCCTGGCAC CTAGCCCCGG TTCCTGGACT
      721 AACTTGTCCC ACTTAGATGG CAATCTGTCC GACCCATGCG GTCCGAACCG CACCGAGCTA
      781 GGGGGGAGCG ACAGCCGGTG CCCTCCGACT GGGAGCCCCT CCATGATCAC GGCTGTCACC
      841 ATCATGGCCC TCTACTCCAT CGTGTGCGTG GTGGGGCTCT TTGGAAACTT CCTGGTCATG
      901 TATGTGATCA TCAGATACAC CAAAATGAAG ACTGCCACCA ATATCTACAT TTTCAATCTT
      961 GCTCTGGCAG ATGCCTTAGC TACCAGTACT CTGCCCTTCC AGAGTGTAAA TTACCTAATG
     1021 GGAACATGGC CTTTTGGAAC CATCCTTTGC AAGATTGTGA TCTCTATAGA TTACTACAAC
     1081 ATGTTCACCA GCATATTCAC CCTCTGCACC ATGAGTGTGG ATCGTTATAT TGCAGTCTGC
     1141 CACCCTGTCA AGGCCCTGGA TTTCCGTACT CCCCGAAATG CCAAAATTGT CAATGTCTGC
     1201 AACTGGATCC TGTCTTCTGC CATTGGTCTG CCTGTAATGT TCATGGCAAC AACAAAATAC
     1261 AGACATGGCT CCATAGATTG TACGCTAACA TTCTCTCACC CAACCTGGTA CTGGGAGAAC
     1321 CTACTGAAGA TCTGTGTCTT CATCTTTGCC TTCATCATGC CTGTCCTCAT AATTACAGTG
     1381 TGTTATGGAC TGATGATCTT ACGCCTCAAG AGTGTCCGCA TGCTGTCAGG CTCCAAAGAA
     1441 AAGGATAGGA ACCTGCGGAG GATCACCAGG ATGGTGCTGG TGGTTGTGGC TGTATTCATT
     1501 GTCTGCTGGA CTCCCATTCA CATTTATGTC ATTATTAAAG CCTTGATCAC TATCCCAGAA
     1561 ACTACTTTCC AAACAGTTTC ATGGCACTTC TGCATTGCTC TAGGTTACAC AAACAGCTGC
     1621 CTGAACCCAG TCCTGTATGC GTTTCTGGAT GAAAACTTCA AACGATGCTT CAGAGAATTC
     1681 TGTATCCCAA CCTCCTCCAC GATTGAGCAA CAAAACTCCA CTCGAATTCG TCAGAACACT
     1741 AGAGACCACC CCTCCACCGC CAACACAGTG GACAGAACAA ACCATCAGCT GGAAAATCTG
     1801 GAAGCAGAAA CAGCTCCGTT GCCCTAACTG GGTCTTGCTG CAGTCGACGG TACCGCGGGC
     1861 CCGGGATCCG CCCCTCTCCC TCCCCCCCCC CTAACGTTAC TGGCCGAAGC CGCTTGGAAT
     1921 AAGGCCGGTG TGCGTTTGTC TATATGTTAT TTTCCACCAT ATTGCCGTCT TTTGGCAATG
     1981 TGAGGGCCCG GAAACCTGGC CCTGTCTTCT TGACGAGCAT TCCTAGGGGT CTTTCCCCTC
     2041 TCGCCAAAGG AATGCAAGGT CTGTTGAATG TCGTGAAGGA AGCAGTTCCT CTGGAAGCTT
     2101 CTTGAAGACA AACAACGTCT GTAGCGACCC TTTGCAGGCA GCGGAACCCC CCACCTGGCG
     2161 ACAGGTGCCT CTGCGGCCAA AAGCCACGTG TATAAGATAC ACCTGCAAAG GCGGCACAAC
     2221 CCCAGTGCCA CGTTGTGAGT TGGATAGTTG TGGAAAGAGT CAAATGGCTC TCCTCAAGCG
     2281 TATTCAACAA GGGGCTGAAG GATGCCCAGA AGGTACCCCA TTGTATGGGA TCTGATCTGG
     2341 GGCCTCGGTG CACATGCTTT ACATGTGTTT AGTCGAGGTT AAAAAAACGT CTAGGCCCCC
     2401 CGAACCACGG GGACGTGGTT TTCCTTTGAA AAACACGATG ATAATATGGC CACAACCATG
     2461 GTGAGCAAGG GCGAGGAGCT GTTCACCGGG GTGGTGCCCA TCCTGGTCGA GCTGGACGGC
     2521 GACGTAAACG GCCACAAGTT CAGCGTGTCC GGCGAGGGCG AGGGCGATGC CACCTACGGC
     2581 AAGCTGACCC TGAAGTTCAT CTGCACCACC GGCAAGCTGC CCGTGCCCTG GCCCACCCTC
     2641 GTGACCACCC TGACCTACGG CGTGCAGTGC TTCAGCCGCT ACCCCGACCA CATGAAGCAG
     2701 CACGACTTCT TCAAGTCCGC CATGCCCGAA GGCTACGTCC AGGAGCGCAC CATCTTCTTC
     2761 AAGGACGACG GCAACTACAA GACCCGCGCC GAGGTGAAGT TCGAGGGCGA CACCCTGGTG
     2821 AACCGCATCG AGCTGAAGGG CATCGACTTC AAGGAGGACG GCAACATCCT GGGGCACAAG
     2881 CTGGAGTACA ACTACAACAG CCACAACGTC TATATCATGG CCGACAAGCA GAAGAACGGC
     2941 ATCAAGGTGA ACTTCAAGAT CCGCCACAAC ATCGAGGACG GCAGCGTGCA GCTCGCCGAC
     3001 CACTACCAGC AGAACACCCC CATCGGCGAC GGCCCCGTGC TGCTGCCCGA CAACCACTAC
     3061 CTGAGCACCC AGTCCGCCCT GAGCAAAGAC CCCAACGAGA AGCGCGATCA CATGGTCCTG
     3121 CTGGAGTTCG TGACCGCCGC CGGGATCACT CTCGGCATGG ACGAGCTGTA CAAGTAAAGC
     3181 GGCCGCGACT CTAGATCATA ATCAGCCATA CCACATTTGT AGAGGTTTTA CTTGCTTTAA
```

```
3241 AAAACCTCCC ACACCTCCCC CTGAACCTGA AACATAAAAT GAATGCAATT GTTGTTGTTA
3301 ACTTGTTTAT TGCAGCTTAT AATGGTTACA AATAAAGCAA TAGCATCACA AATTTCACAA
3361 ATAAAGCATT TTTTTCACTG CATTCTAGTT GTGGTTTGTC CAAACTCATC AATGTATCTT
3421 AAGGCGTAAA TTGTAAGCGT TAATATTTTG TTAAAATTCG CGTTAAATTT TTGTTAAATC
3481 AGCTCATTTT TTAACCAATA GGCCGAAATC GGCAAAATCC CTTATAAATC AAAAGAATAG
3541 ACCGAGATAG GGTTGAGTGT TGTTCCAGTT TGGAACAAGA GTCCACTATT AAAGAACGTG
3601 GACTCCAACG TCAAAGGGCG AAAAACCGTC TATCAGGGCG ATGGCCCACT ACGTGAACCA
3661 TCACCCTAAT CAAGTTTTTT GGGGTCGAGG TGCCGTAAAG CACTAAATCG GAACCCTAAA
3721 GGGAGCCCCC GATTTAGAGC TTGACGGGGA AAGCCGGCGA ACGTGGCGAG AAAGGAAGGG
3781 AAGAAAGCGA AAGGAGCGGG CGCTAGGGCG CTGGCAAGTG TAGCGGTCAC GCTGCGCGTA
3841 ACCACCACAC CCGCCGCGCT TAATGCGCCG CTACAGGGCG CGTCAGGTGG CACTTTTCGG
3901 GGAAATGTGC GCGGAACCCC TATTTGTTTA TTTTTCTAAA TACATTCAAA TATGTATCCG
3961 CTCATGAGAC AATAACCCTG ATAAATGCTT CAATAATATT GAAAAAGGAA GAGTCCTGAG
4021 GCGGAAAGAA CCAGCTGTGG AATGTGTGTC AGTTAGGGTG TGGAAAGTCC CCAGGCTCCC
4081 CAGCAGGCAG AAGTATGCAA AGCATGCATC TCAATTAGTC AGCAACCAGG TGTGGAAAGT
4141 CCCCAGGCTC CCCAGCAGGC AGAAGTATGC AAAGCATGCA TCTCAATTAG TCAGCAACCA
4201 TAGTCCCGCC CCTAACTCCG CCCATCCCGC CCCTAACTCC GCCCAGTTCC GCCCATTCTC
4261 CGCCCCATGG CTGACTAATT TTTTTTATTT ATGCAGAGGC CGAGGCCGCC TCGGCCTCTG
4321 AGCTATTCCA GAAGTAGTGA GGAGGCTTTT TTGGAGGCCT AGGCTTTTGC AAAGATCGAT
4381 CAAGAGACAG GATGAGGATC GTTTCGCATG ATTGAACAAG ATGGATTGCA CGCAGGTTCT
4441 CCGGCCGCTT GGGTGGAGAG GCTATTCGGC TATGACTGGG CACAACAGAC AATCGGCTGC
4501 TCTGATGCCG CCGTGTTCCG GCTGTCAGCG CAGGGGCGCC CGGTTCTTTT TGTCAAGACC
4561 GACCTGTCCG GTGCCCTGAA TGAACTGCAA GACGAGGCAG CGCGGCTATC GTGGCTGGCC
4621 ACGACGGGCG TTCCTTGCGC AGCTGTGCTC GACGTTGTCA CTGAAGCGGG AAGGGACTGG
4681 CTGCTATTGG GCGAAGTGCC GGGGCAGGAT CTCCTGTCAT CTCACCTTGC TCCTGCCGAG
4741 AAAGTATCCA TCATGGCTGA TGCAATGCGG CGGCTGCATA CGCTTGATCC GGCTACCTGC
4801 CCATTCGACC ACCAAGCGAA ACATCGCATC GAGCGAGCAC GTACTCGGAT GGAAGCCGGT
4861 CTTGTCGATC AGGATGATCT GGACGAAGAG CATCAGGGGC TCGCGCCAGC CGAACTGTTC
4921 GCCAGGCTCA AGGCGAGCAT GCCCGACGGC GAGGATCTCG TCGTGACCCA TGGCGATGCC
4981 TGCTTGCCGA ATATCATGGT GGAAAATGGC CGCTTTTCTG GATTCATCGA CTGTGGCCGG
5041 CTGGGTGTGG CGGACCGCTA TCAGGACATA GCGTTGGCTA CCCGTGATAT TGCTGAAGAG
5101 CTTGGCGGCG AATGGGCTGA CCGCTTCCTC GTGCTTTACG GTATCGCCGC TCCCGATTCG
5161 CAGCGCATCG CCTTCTATCG CCTTCTTGAC GAGTTCTTCT GAGCGGGACT CTGGGGTTCG
5221 AAATGACCGA CCAAGCGACG CCCAACCTGC CATCACGAGA TTTCGATTCC ACCGCCGCCT
5281 TCTATGAAAG GTTGGGCTTC GGAATCGTTT TCCGGGACGC CGGCTGGATG ATCCTCCAGC
5341 GCGGGGATCT CATGCTGGAG TTCTTCGCCC ACCCTAGGGG GAGGCTAACT GAAACACGGA
5401 AGGAGACAAT ACCGGAAGGA ACCCGCGCTA TGACGGCAAT AAAAAGACAG AATAAAACGC
5461 ACGGTGTTGG GTCGTTTGTT CATAAACGCG GGGTTCGGTC CCAGGGCTGG CACTCTGTCG
5521 ATACCCCACC GAGACCCCAT TGGGGCCAAT ACGCCCGCGT TTCTTCCTTT TCCCCACCCC
5581 ACCCCCCAAG TTCGGGTGAA GGCCCAGGGC TCGCAGCCAA CGTCGGGGCG GCAGGCCCTG
5641 CCATAGCCTC AGGTTACTCA TATATACTTT AGATTGATTT AAAACTTCAT TTTTAATTTA
5701 AAAGGATCTA GGTGAAGATC CTTTTTGATA ATCTCATGAC CAAAATCCCT TAACGTGAGT
5761 TTTCGTTCCA CTGAGCGTCA GACCCCGTAG AAAAGATCAA AGGATCTTCT TGAGATCCTT
5821 TTTTTCTGCG CGTAATCTGC TGCTTGCAAA CAAAAAAACC ACCGCTACCA GCGGTGGTTT
5881 GTTTGCCGGA TCAAGAGCTA CCAACTCTTT TTCCGAAGGT AACTGGCTTC AGCAGAGCGC
5941 AGATACCAAA TACTGTCCTT CTAGTGTAGC CGTAGTTAGG CCACCACTTC AAGAACTCTG
6001 TAGCACCGCC TACATACCTC GCTCTGCTAA TCCTGTTACC AGTGGCTGCT GCCAGTGGCG
6061 ATAAGTCGTG TCTTACCGGG TTGGACTCAA GACGATAGTT ACCGGATAAG GCGCAGCGGT
6121 CGGGCTGAAC GGGGGGTTCG TGCACACAGC CCAGCTTGGA GCGAACGACC TACACCGAAC
6181 TGAGATACCT ACAGCGTGAG CTATGAGAAA GCGCCACGCT TCCCGAAGGG AGAAAGGCGG
6241 ACAGGTATCC GGTAAGCGGC AGGGTCGGAA CAGGAGAGCG CACGAGGGAG CTTCCAGGGG
6301 GAAACGCCTG GTATCTTTAT AGTCCTGTCG GGTTTCGCCA CCTCTGACTT GAGCGTCGAT
6361 TTTTGTGATG CTCGTCAGGG GGGCGGAGCC TATGGAAAAA CGCCAGCAAC GCGGCCTTTT
6421 TACGGTTCCT GGCCTTTTGC TGGCCTTTTG CTCACATGTT CTTTCCTGCG TTATCCCCTG
6481 ATTCTGTGGA TAACCGTATT ACCGCCATGC AT
//
```
