# Supplementary material for: Functional Characteristics of the Naked Mole Rat μ-Opioid Receptor
Source: PLoS One. 2013 Nov 27;8(11):e79121. doi: 10.1371/journal.pone.0079121 (PMC3842265; doi:10.1371/journal.pone.0079121)
Supplement: Text S3 — NMR pCMV-oprm1eGFP Vector map. (PDF) [file pone.0079121.s007.pdf]

pCMV-NMR *oprm1*eGFP Vector map

LOCUS   pCMV-NMR *oprm1*eGFP   5879 bp ds-DNA   circular
ORGANISM   Naked mole rat (*Heterocephalus glaber*)
COMMENT   IRES was excised from pCMV-NMR *oprm1*-IRES-eGFP
FEATURES   Location/Qualifiers
     CMV-Promotor   10..589
     NMR *oprm1*   625..1824
     eGFP   1825..2544
     NMR delete IRES primer   1811..1838
     SV40_promoter   3556..3735
     Kan/neoR   3775..4569

ORIGIN
1 TAGTTATTAA TAGTAATCAA TTACGGGGTC ATTAGTTCAT AGCCCATATA TGGAGTTCCG
61 CGTTACATAA CTTACGGTAA ATGGCCCGCC TGGCTGACCG CCCAACGACC CCCGCCCATT
121 GACGTCAATA ATGACGTATG TTCCCATAGT AACGCCAATA GGGACTTTCC ATTGACGTCA
181 ATGGGTGGAG TATTTACGGT AAACTGCCCA CTTGGCAGTA CATCAAGTGT ATCATATGCC
241 AAGTACGCCC CCTATTGACG TCAATGACGG TAAATGGCCC GCCTGGCATT ATGCCCAGTA
301 CATGACCTTA TGGGACTTTC CTACTTGGCA GTACATCTAC GTATTAGTCA TCGCTATTAC
361 CATGGTGATG CGGTTTTGGC AGTACATCAA TGGGCGTGGA TAGCGGTTTG ACTCACGGGG
421 ATTTCCAAGT CTCCACCCCA TTGACGTCAA TGGGAGTTTG TTTTGGCACC AAAATCAACG
481 GGACTTTCCA AAATGTCGTA ACAACTCCGC CCCATTGACG CAAATGGGCG GTAGGCGTGT
541 ACGGTGGGAG GTCTATATAA GCAGAGCTGG TTTAGTGAAC CGTCAGATCC GCTAGCGCTA
601 CCGGACTCAG ATCTCGAGAG CACC**ATG**GAC AGCAGTGTCC TGCCCGGGAA CGCGGGCAAT
661 TGCACCGATC CCTTCGCGCA ATCCAGTTGC TCCCTGGCAC CTAGCCCCGG TTCCTGGACT
721 AACTTGTCCC ACTTAGATGG CAATCTGTCC GACCCATGCG GTCCGAACCG CACCGAGCTA
781 GGGGGGAGCG ACAGCCGGTG CCCTCCGACT GGGAGCCCCT CCATGATCAC GGCTGTCACC
841 ATCATGGCCC TCTACTCCAT CGTGTGCGTG GTGGGGCTCT TTGGAAACTT CCTGGTCATG
901 TATGTGATCA TCAGATACAC CAAAATGAAG ACTGCCACCA ATATCTACAT TTTCAATCTT
961 GCTCTGGCAG ATGCCTTAGC TACCAGTACT CTGCCCTTCC AGAGTGTAAA TTACCTAATG
1021 GGAACATGGC CTTTTGGAAC CATCCTTTGC AAGATTGTGA TCTCTATAGA TTACTACAAC
1081 ATGTTCACCA GCATATTCAC CCTCTGCACC ATGAGTGTGG ATCGTTATAT TGCAGTCTGC
1141 CACCCTGTCA AGGCCCTGGA TTTCCGTACT CCCCGAAATG CCAAAATTGT CAATGTCTGC
1201 AACTGGATCC TGTCTTCTGC CATTGGTCTG CCTGTAATGT TCATGGCAAC AACAAAATAC
1261 AGACATGGCT CCATAGATTG TACGCTAACA TTCTCTCACC CAACCTGGTA CTGGGAGAAC
1321 CTACTGAAGA TCTGTGTCTT CATCTTTGCC TTCATCATGC CTGTCCTCAT AATTACAGTG
1381 TGTTATGGAC TGATGATCTT ACGCCTCAAG AGTGTCCGCA TGCTGTCAGG CTCCAAAGAA
1441 AAGGATAGGA ACCTGCGGAG GATCACCAGG ATGGTGCTGG TGGTTGTGGC TGTATTCATT
1501 GTCTGCTGGA CTCCCATTCA CATTTATGTC ATTATTAAAG CCTTGATCAC TATCCCAGAA
1561 ACTACTTTCC AAACAGTTTC ATGGCACTTC TGCATTGCTC TAGGTTACAC AAACAGCTGC
1621 CTGAACCCAG TCCTGTATGC GTTTCTGGAT GAAAACTTCA AACGATGCTT CAGAGAATTC
1681 TGTATCCCAA CCTCCTCCAC GATTGAGCAA CAAAACTCCA CTCGAATTCG TCAGAACACT
1741 AGAGACCACC CCTCCACCGC CAACACAGTG GACAGAACAA ACCATCAGCT GGAAAATCTG
1801 GAAGCAGAAA CAGCTCCGTT GCCC**ATG**GTG AGCAAGGGCG AGGAGCTGTT CACCGGGGTG
1861 GTGCCCATCC TGGTCGAGCT GGACGGCGAC GTAAACGGCC ACAAGTTCAG CGTGTCCGGC
1921 GAGGGCGAGG GCGATGCCAC CTACGGCAAG CTGACCCTGA AGTTCATCTG CACCACCGGC
1981 AAGCTGCCCG TGCCCTGGCC CACCCTCGTG ACCACCCTGA CCTACGGCGT GCAGTGCTTC
2041 AGCCGCTACC CCGACCACAT GAAGCAGCAC GACTTCTTCA AGTCCGCCAT GCCCGAAGGC
2101 TACGTCCAGG AGCGCACCAT CTTCTTCAAG GACGACGGCA ACTACAAGAC CCGCGCCGAG
2161 GTGAAGTTCG AGGGCGACAC CCTGGTGAAC CGCATCGAGC TGAAGGGCAT CGACTTCAAG
2221 GAGGACGGCA ACATCCTGGG GCACAAGCTG GAGTACAACT ACAACAGCCA CAACGTCTAT
2281 ATCATGGCCG ACAAGCAGAA GAACGGCATC AAGGTGAACT TCAAGATCCG CCACAACATC
2341 GAGGACGGCA GCGTGCAGCT CGCCGACCAC TACCAGCAGA ACACCCCCAT CGGCGACGGC
2401 CCCGTGCTGC TGCCCGACAA CCACTACCTG AGCACCCAGT CCGCCCTGAG CAAAGACCCC
2461 AACGAGAAGC GCGATCACAT GGTCCTGCTG GAGTTCGTGA CCGCCGCCGG GATCACTCTC
2521 GGCATGGACG AGCTGTACAA G**TAA**AGCGGC CGCGACTCTA GATCATAATC AGCCATACCA
2581 CATTTGTAGA GGTTTTACTT GCTTTAAAAA ACCTCCCACA CCTCCCCCTG AACCTGAAAC
2641 ATAAAATGAA TGCAATTGTT GTTGTTAACT TGTTTATTGC AGCTTATAAT GGTTACAAAT
2701 AAAGCAATAG CATCACAAAT TTCACAAATA AAGCATTTTT TTCACTGCAT TCTAGTTGTG
2761 GTTTGTCCAA ACTCATCAAT GTATCTTAAG GCGTAAATTG TAAGCGTTAA TATTTTGTTA
2821 AAATTCGCGT TAAATTTTTG TTAAATCAGC TCATTTTTTA ACCAATAGGC CGAAATCGGC
2881 AAAATCCCTT ATAAATCAAA AGAATAGACC GAGATAGGGT TGAGTGTTGT TCCAGTTTGG
2941 AACAAGAGTC CACTATTAAA GAACGTGGAC TCCAACGTCA AAGGGCGAAA AACCGTCTAT

```
3001 CAGGGCGATG GCCCACTACG TGAACCATCA CCCTAATCAA GTTTTTTGGG GTCGAGGTGC
3061 CGTAAAGCAC TAAATCGGAA CCCTAAAGGG AGCCCCCGAT TTAGAGCTTG ACGGGGAAAG
3121 CCGGCGAACG TGGCGAGAAA GGAAGGGAAG AAAGCGAAAG GAGCGGGCGC TAGGGCGCTG
3181 GCAAGTGTAG CGGTCACGCT GCGCGTAACC ACCACACCCG CCGCGCTTAA TGCGCCGCTA
3241 CAGGGCGCGT CAGGTGGCAC TTTTCGGGGA AATGTGCGCG GAACCCCTAT TTGTTTATTT
3301 TTCTAAATAC ATTCAAATAT GTATCCGCTC ATGAGACAAT AACCCTGATA AATGCTTCAA
3361 TAATATTGAA AAAGGAAGAG TCCTGAGGCG GAAAGAACCA GCTGTGGAAT GTGTGTCAGT
3421 TAGGGTGTGG AAAGTCCCCA GGCTCCCCAG CAGGCAGAAG TATGCAAAGC ATGCATCTCA
3481 ATTAGTCAGC AACCAGGTGT GGAAAGTCCC CAGGCTCCCC AGCAGGCAGA AGTATGCAAA
3541 GCATGCATCT CAATTAGTCA GCAACCATAG TCCCGCCCCT AACTCCGCCC ATCCCGCCCC
3601 TAACTCCGCC CAGTTCCGCC CATTCTCCGC CCCATGGCTG ACTAATTTTT TTTATTTATG
3661 CAGAGGCCGA GGCCGCCTCG GCCTCTGAGC TATTCCAGAA GTAGTGAGGA GGCTTTTTTG
3721 GAGGCCTAGG CTTTTGCAAA GATCGATCAA GAGACAGGAT GAGGATCGTT TCGCATGATT
3781 GAACAAGATG GATTGCACGC AGGTTCTCCG GCCGCTTGGG TGGAGAGGCT ATTCGGCTAT
3841 GACTGGGCAC AACAGACAAT CGGCTGCTCT GATGCCGCCG TGTTCCGGCT GTCAGCGCAG
3901 GGGCGCCCGG TTCTTTTTGT CAAGACCGAC CTGTCCGGTG CCCTGAATGA ACTGCAAGAC
3961 GAGGCAGCGC GGCTATCGTG GCTGGCCACG ACGGGCGTTC CTTGCGCAGC TGTGCTCGAC
4021 GTTGTCACTG AAGCGGGAAG GGACTGGCTG CTATTGGGCG AAGTGCCGGG GCAGGATCTC
4081 CTGTCATCTC ACCTTGCTCC TGCCGAGAAA GTATCCATCA TGGCTGATGC AATGCGGCGG
4141 CTGCATACGC TTGATCCGGC TACCTGCCCA TTCGACCACC AAGCGAAACA TCGCATCGAG
4201 CGAGCACGTA CTCGGATGGA AGCCGGTCTT GTCGATCAGG ATGATCTGGA CGAAGAGCAT
4261 CAGGGGCTCG CGCCAGCCGA ACTGTTCGCC AGGCTCAAGG CGAGCATGCC CGACGGCGAG
4321 GATCTCGTCG TGACCCATGG CGATGCCTGC TTGCCGAATA TCATGGTGGA AAATGGCCGC
4381 TTTTCTGGAT TCATCGACTG TGGCCGGCTG GGTGTGGCGG ACCGCTATCA GGACATAGCG
4441 TTGGCTACCC GTGATATTGC TGAAGAGCTT GGCGGCGAAT GGGCTGACCG CTTCCTCGTG
4501 CTTTACGGTA TCGCCGCTCC CGATTCGCAG CGCATCGCCT TCTATCGCCT TCTTGACGAG
4561 TTCTTCTGAG CGGGACTCTG GGGTTCGAAA TGACCGACCA AGCGACGCCC AACCTGCCAT
4621 CACGAGATTT CGATTCCACC GCCGCCTTCT ATGAAAGGTT GGGCTTCGGA ATCGTTTTCC
4681 GGGACGCCGG CTGGATGATC CTCCAGCGCG GGGATCTCAT GCTGGAGTTC TTCGCCCACC
4741 CTAGGGGGAG GCTAACTGAA ACACGGAAGG AGACAATACC GGAAGGAACC CGCGCTATGA
4801 CGGCAATAAA AAGACAGAAT AAAACGCACG GTGTTGGGTC GTTTGTTCAT AAACGCGGGG
4861 TTCGGTCCCA GGGCTGGCAC TCTGTCGATA CCCCACCGAG ACCCCATTGG GGCCAATACG
4921 CCCGCGTTTC TTCCTTTTCC CCACCCCACC CCCCAAGTTC GGGTGAAGGC CCAGGGCTCG
4981 CAGCCAACGT CGGGGCGGCA GGCCCTGCCA TAGCCTCAGG TTACTCATAT ATACTTTAGA
5041 TTGATTTAAA ACTTCATTTT TAATTTAAAA GGATCTAGGT GAAGATCCTT TTTGATAATC
5101 TCATGACCAA AATCCCTTAA CGTGAGTTTT CGTTCCACTG AGCGTCAGAC CCCGTAGAAA
5161 AGATCAAAGG ATCTTCTTGA GATCCTTTTT TTCTGCGCGT AATCTGCTGC TTGCAAACAA
5221 AAAAACCACC GCTACCAGCG GTGGTTTGTT TGCCGGATCA AGAGCTACCA ACTCTTTTTC
5281 CGAAGGTAAC TGGCTTCAGC AGAGCGCAGA TACCAAATAC TGTCCTTCTA GTGTAGCCGT
5341 AGTTAGGCCA CCACTTCAAG AACTCTGTAG CACCGCCTAC ATACCTCGCT CTGCTAATCC
5401 TGTTACCAGT GGCTGCTGCC AGTGGCGATA AGTCGTGTCT TACCGGGTTG GACTCAAGAC
5461 GATAGTTACC GGATAAGGCG CAGCGGTCGG GCTGAACGGG GGGTTCGTGC ACACAGCCCA
5521 GCTTGGAGCG AACGACCTAC ACCGAACTGA GATACCTACA GCGTGAGCTA TGAGAAAGCG
5581 CCACGCTTCC CGAAGGGAGA AAGGCGGACA GGTATCCGGT AAGCGGCAGG GTCGGAACAG
5641 GAGAGCGCAC GAGGGAGCTT CCAGGGGGAA ACGCCTGGTA TCTTTATAGT CCTGTCGGGT
5701 TTCGCCACCT CTGACTTGAG CGTCGATTTT TGTGATGCTC GTCAGGGGGG CGGAGCCTAT
5761 GGAAAAACGC CAGCAACGCG GCCTTTTTAC GGTTCCTGGC CTTTTGCTGG CCTTTTGCTC
5821 ACATGTTCTT TCCTGCGTTA TCCCCTGATT CTGTGGATAA CCGTATTACC GCCATGCAT
//
```
